# In-Situ Dynamic Response Measurement for Damage Quantification of 3D Printed ABS Cantilever Beam under Thermomechanical Load

**DOI:** 10.3390/polym11122079

**Published:** 2019-12-12

**Authors:** Hamzah Baqasah, Feiyang He, Behzad A. Zai, Muhammad Asif, Kamran A. Khan, Vijay K. Thakur, Muhammad A. Khan

**Affiliations:** 1 School of Aerospace, Transport and Manufacturing, Cranfield University, Cranfield MK43 0AL, UK; hamzah.baqasah@gmail.com (H.B.); feiyang.he@cranfield.ac.uk (F.H.); 2 Department of Engineering Sciences, PN Engineering College, National University of Sciences and Technology (NUST), Karachi 75350, Pakistanmasif@aut.ac.nz (M.A.); 3 Department of Aerospace Engineering, Khalifa University, Abu Dhabi 127788, UAE; kamran.khan@ku.ac.ae

**Keywords:** acrylonitrile butadiene styrene, dynamic response, fatigue, crack propagation, fused deposition modeling, FDM, fused filament fabrication, modal analysis, cantilever beam

## Abstract

Acrylonitrile butadiene styrene (ABS) offers good mechanical properties and is effective in use to make polymeric structures for industrial applications. It is one of the most common raw material used for printing structures with fused deposition modeling (FDM). However, most of its properties and behavior are known under quasi-static loading conditions. These are suitable to design ABS structures for applications that are operated under static or dead loads. Still, comprehensive research is required to determine the properties and behavior of ABS structures under dynamic loads, especially in the presence of temperature more than the ambient. The presented research was an effort mainly to provide any evidence about the structural behavior and damage resistance of ABS material if operated under dynamic load conditions coupled with relatively high-temperature values. A non-prismatic fixed-free cantilever ABS beam was used in this study. The beam specimens were manufactured with a 3D printer based on FDM. A total of 190 specimens were tested with a combination of different temperatures, initial seeded damage or crack, and crack location values. The structural dynamic response, crack propagation, crack depth quantification, and their changes due to applied temperature were investigated by using analytical, numerical, and experimental approaches. In experiments, a combination of the modal exciter and heat mats was used to apply the dynamic loads on the beam structure with different temperature values. The response measurement and crack propagation behavior were monitored with the instrumentation, including a 200× microscope, accelerometer, and a laser vibrometer. The obtained findings could be used as an in-situ damage assessment tool to predict crack depth in an ABS beam as a function of dynamic response and applied temperature.

## 1. Introduction

Additive manufacturing (AM) has become increasingly important to produce high-quality functional components. In the past, AM was mainly used for prototyping, but nowadays, it is being utilized to manufacture automotive and aerospace parts due to its high precision and surface quality [1,2]. Fused deposited modeling (FDM) is a very common AM technique that is mainly limited to polymer materials. FDM involves heating near the melting point to have a liquid-like material layer for 3D printing of a geometry. The heated material is extruded from a computer-controlled nozzle with high accuracy to produce complex geometries. The process is carried on the layer by layer, and each layer is bonded with the former by means of thermal diffusion that occurs due to a relatively high-temperature environment [3].

3D printed polymeric structures have now gained a lot of attention from industrial and academic researchers [4]. Recent investigations show that these structures can be reinforced with carbon fibers and carbon nanomaterials and can replace the existing costly structural components in automobile and aircraft applications [5,6]. Nikolova and Chavali, and Stratton et al. recently reviewed the application of 3D bio-printed structures for restoration and reconstruction of different anatomical defects of complex organs and functional tissues [7,8]. Agu et al. investigated the strength of 3D printed polylactic acid (PLA) to manufacture components exposed to high strain-rate/impact events during their design life [9]. They claimed that shear strength with increasing impact stress had been measured for the very first time for any PLA-based 3D printed structure. 3D printed macroscopic structures have also been employed to mimic the complex gestures of human hands in the application of soft robotics [10]. Due to this wide spectrum of usage, academic research studies have investigated the performance of the 3D printed polymeric structures under different operational conditions. Most of their properties and behavior are known under quasi-static loading conditions. These are suitable to design polymeric structures for applications that are operated under quasi-static or dead loads. Qin-Zhi, et al. [11] studied crack propagation under static load to observe the crack mechanics in polymers. Marcos Lugo, et al. mentioned that multi-stage fatigue (MSF) modeling could be used to predict crack initiation, crack growth, and different fatigue regime [12]. However, still, comprehensive research is required to determine the properties and behavior of these structures under dynamic loads, especially in the presence of temperature more than the ambient.

The material of the polymeric structure selected in the presented study was acrylonitrile butadiene styrene (ABS), which is one of the most common raw material used for printing structures with fused deposition modeling (FDM). ABS properties are compatible with the FDM process, such as heat resistance and low-temperature impact resistance [13]. ABS also offers good chemical and corrosion resistance, high toughness, and impact strength. But equally, it has limitations in use due to its complex morphology and composition [14]. Printing factors, such as the orientation of layers and filling density, can also influence the mechanical properties of printed ABS structure. Whereas the machine parameters, such as nozzle diameter, can affect the accuracy of fabricated samples [3,13,15].

A few researchers have investigated crack mechanics of 3D printed ABS structures using customized experimental setups. At ambient conditions, natural frequency-based methods have been used to quantify cracks in a beam. Behzad et al. tested metallic and polymeric structures under dynamic loads and established a correlation for crack depth prediction [16]. Hanyin studied 3D printed ABS and performed mechanical characterization, including tensile, creep, and fatigue strength [17]. Changes in mechanical properties of ABS have been observed due to different operating temperatures [18]. Disorder and stretch at the atomic scale have been concluded as the main causes for these changes. Different crack growth behavior in ABS at elevated and ambient temperatures has been observed mainly due to an increase in plasticity at the tip of the crack [19]. Mai [20], Martin [21], and Kim [22] also concluded that decreasing temperature could decrease crack growth in polymers. Kim reported that elevated temperatures could induce chain disentanglement and chain slippage and hence decrease the crack resistance in polymers under thermo-mechanical loads [23]. Still, a mathematical relationship that can describe crack growth in ABS in the presence of dynamic loads with high operating temperatures (as compared to ambient) is not available.

In this paper, an effort was made mainly to develop the required mathematical relation between the structural behavior and crack resistance of the ABS beam structure if operated under dynamic load conditions coupled with relatively high-temperature values. Detecting damages by using the dynamic response of the structure is a very effective non-destructive testing technique [24,25]. Due to this reason, the structural dynamic response was used to investigate crack growth, quantification, and location of the mentioned ABS beam. The structural dynamic response, crack propagation, crack depth quantification, and their changes due to applied temperature were investigated by using analytical, numerical, and experimental approaches. In experiments, a combination of the modal exciter and heat mats was used to apply the dynamic loads on the beam structure with different temperature values. The response measurement and crack propagation behavior were monitored with the instrumentation, including a 200× microscope, accelerometer, and a laser vibrometer. The obtained findings could be used as an in-situ damage assessment tool to predict crack depth in an ABS beam as a function of dynamic response and applied temperature.

## 2. Materials and Methods

This section presents all the three approaches (experimental, analytical, and numerical) to evaluate the relationship between the dynamic response and crack depth/location for 3D printing ABS specimen under thermomechanical dynamic loads.

### 2.1. Experimental Method

**Figure 1:**
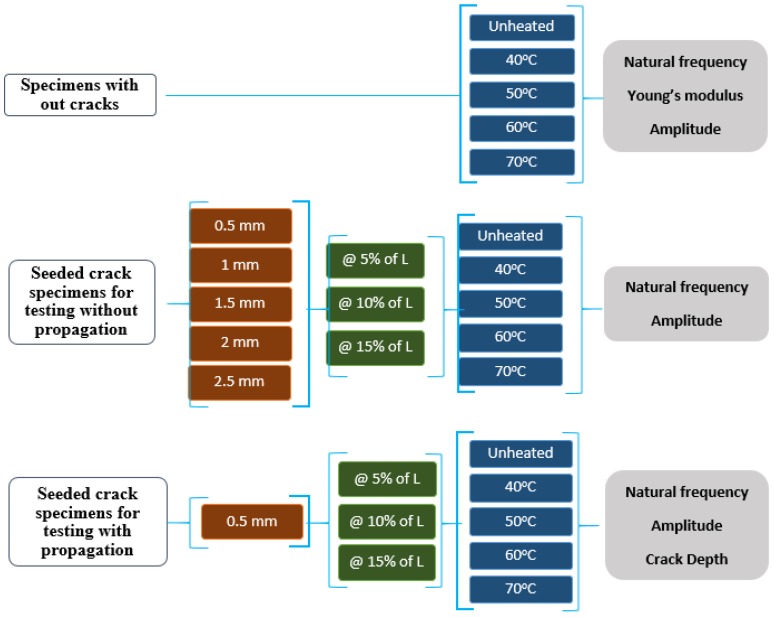
The comprehensive schematic diagram for experiments.

A series of tests were conducted, and the dynamic response parameters were measured, including natural frequency and displacement amplitude. Five different temperature values were used during the tests: 25 °C, 40 °C, 50 °C, 60 °C, and 70 °C. In order to maintain stable mechanical properties, the maximum temperature of 343 K (i.e., 70 °C) was chosen, which is approximately 90% of the glass transition temperature (378 K) of ABS. The complete experimental scheme is shown in Figure 1.

The tests were divided into three sets. The first set used 10 specimens without any seeded crack to measure Young’s modulus, amplitude, and natural frequency responses. Five pairs of 2 specimens were tested on different values of temperature ranging from room temperature to 70 °C, as shown in Figure 1. The second set used a total of 150 specimens with an initial seeded crack ranging from 0.5 mm to 2.5 mm. In this set, all the possible combinations of different initial seeded crack depth values, crack location, and operating temperature were tested, as shown in Figure 1. Each of the combinations was tested on a pair of 2 specimens; hence, we had in total of 75 pairs to test for their instant or one-off natural frequency and amplitude responses. We assumed seeded cracks were not propagated during any of these tests, and hence the stiffness of the beam considered not to be changed and influenced on the measured responses. The third set used a total of 30 specimens with an initial seeded crack value of 0.5 mm only. In this set, all the possible combinations of crack location and operating temperature were tested, as shown in Figure 1. Each of the combinations was tested on a pair of 2 specimens; hence, we had in total of 15 pairs to test their natural frequency, amplitude, and crack depth responses while crack was propagating. The propagation allowed a changed in beam stiffness and, hence, influenced the measured responses.

At the beginning of each test, an impact test was performed to identify the natural frequency of the specimens, which is explained in detail in Section 2.1.4. The natural frequency response was measured by a laser vibrometer (i.e., Polytec PDV 100, Coventry, UK). Later, the beam was subjected to a cyclic load at the natural frequency, and its amplitude response was captured by a microscopic camera (i.e., Dino-lite AM4113T, AnMo Electronics Corporation, Taiwan).

#### 2.1.1. Specimen Fabrication

**Figure 2:**
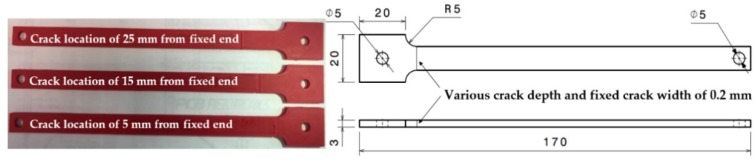
(**Left**) Different crack locations introduced into the specimen; (**Right**) Specimen geometry as designed in SolidWorks.

ABS material of red color was used to make test specimens. The red color was helpful in observing the details of the crack propagation. The specimen was designed as a cantilever beam, and its geometrical dimension is shown in Figure 2. The effective length and the thickness of the beam were fixed 150 mm and 3 mm, respectively, in all the tests. In order to study how the crack location affects the dynamic response of the cracked structure, specimens were fabricated with cracks at three different distances (i.e., 5 mm, 15 mm, and 25 mm) from the fixed end of the beam. Further, long-distance values were not selected because it could shift the high-stress concentration zone from crack location to the fixed end of the beam. This stress concentration shift could produce fracture at the fixed end rather than at the crack location and, hence, could make the observation of crack growth difficult. Five initial seeded crack depths, from 0.5 mm to 2.5 mm with an increment of 0.5 mm, were used. The crack width was fixed as 0.2 mm to get good accuracy during 3D printing.

#### 2.1.2. Printing Set-Up

All specimens were fabricated on the Ultimaker 2 + 3D printer (Ultimaker B.V., Utrecht, The Netherlands). The geometry of the beam was designed in SolidWorks© (Dassault Systemes SolidWorks Corporation, Waltham, MA, USA) and imported into the CURA software for printing preparation.

The main 3D printing parameters are listed in Table 1. These parameters play an important role in the mechanical properties of any printed structure. Brian et al. proposed [2] that the strength of polymer bonding between the neighboring beads in any part limits its mechanical properties. They further suggested that the temperature history of a road at the interface with another road is a crucial variable in determining the quality of the bond. Therefore, an optimum printing path is always critical for increasing the specimen’s strength. Thrimurthulu et al. proposed that the part deposition or building orientation not only affects the structural strength but also effects the build time, dimensional accuracy, and cost of the prototype. An optimum printing path has to trade-off among various contradicting objectives [26]. Furthermore, Zhou et al. investigated the bonding effect for FDM polycarbonate and acrylonitrile-butadiene-styrene composites based on two simplified deposition modes. In their research, the deposition path orientation, which was parallel to the direction of the tensile test, showed better strength than the vertical direction [27]. With this evidence from previous research, ±45° building orientation was used in the fabrication of test specimens. The platform was heated to 80 °C for minimizing the dimensional error due to the contraction. High accuracy of 3D printing made it possible to craft a 0.2 mm wide initially seeded crack. The completed specimen and its details are shown in Figure 2. It was intuitive and convenient to calculate the crack depth by counting the number of layers through which the crack was passed.

#### 2.1.3. Experimental Set-Up

**Figure 3:**
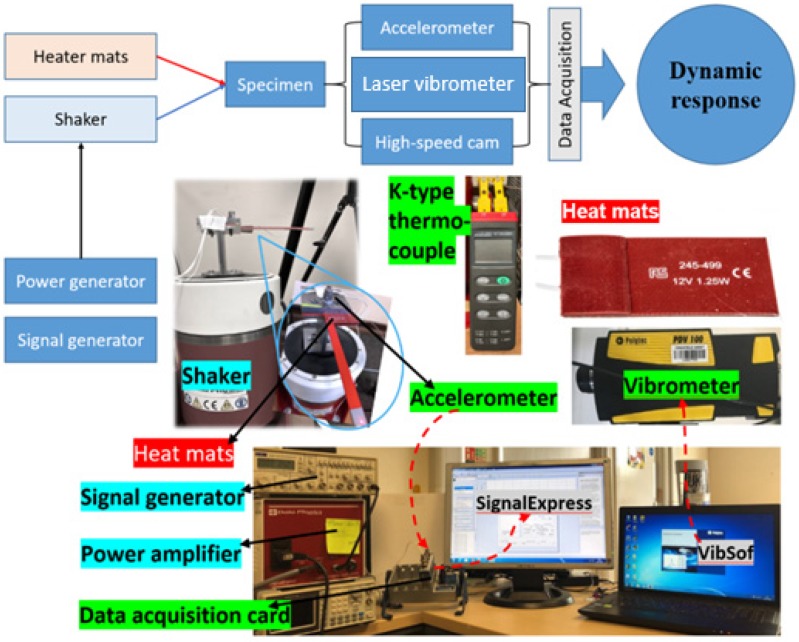
Experiment set-up.

The complete experimental set-up is shown in Figure 3. The set-up can be divided into four parts, including the vibration system, the heating system, the dynamic response data acquisition platform, and crack propagation capture equipment.

The beam was inserted into an ABS holder that ensures the same boundary conditions for all tests and then clamped with two steel plates with four bolts on the top of the exciter’s shaft. Each bolt was fastened with the same number of turns, and the height of the mounting was measured by a digital calliper. No more than 0.5% height’s difference was allowed to obtain constant boundary conditions.

Dynamic load set-up included a signal generator (TG 550), a power amplifier, and a modal exciter or shaker (V55), all made of Data Physics (Data Physics, CA, USA). The shaker was bolted on the floor in order to keep the stable vibration output. The signal generator was set at 5 volts sinusoid signal that provides a 1 mm displacement at the shaker shaft with the help of a power amplifier.

Silicone heating mats and K-type thermocouple (RS Components, Northants, UK) were used to apply and record the temperature. Two silicone heater mats were installed near the fixed end of the specimen, as shown in Figure 3. The lab temperature was set at 25 °C to ensure the same initial conditions. The bench power supply was used to provide adjustable voltages to both the heating mats. For each specimen, apart from continuous heating during the test, the crack area of the specimen was pre-heated and insulated with the desired temperature for 10 min before the test to confirm the constant thermal condition. Two K type thermocouples were mounted between the heating mats and the specimen for continuous temperature monitoring. The voltage applied to the thermocouple was fine-tuned during the test, and the difference between the actual and required temperatures was maintained under 0.2 °C.

Several measurement tools were used for different parameters. The laser vibrometer with Vibosoft© software (Polytec PDV 100, Coventry, UK) was used to measure the approximate natural frequency of the specimen during impact tests. An accelerometer was mounted at the fixed end of the specimen to record the excitation frequency during the test. The displacement amplitude at the beam tip was continuously monitored with the Dino-Lite microscopic camera. Accelerometer measurements were acquired with NI-9174 DAQ card and Signal Express software. Crack depths were measured with the mentioned camera at a magnification of 200×. Besides this, as mentioned before, the cracked printing layers were also counted for a reference.

#### 2.1.4. E-Modulus Measurement

The Young’s modulus of the ABS material under different temperatures was measured using a dynamic mechanical analyzer (DMA Q800, TA instruments, Delware, USA). Two rectangle specimens with the same 3D printing parameters were tested within a temperature range from 30 °C to 70 °C at a frequency of 1 Hz. An empirical relation between Young’s modulus and the temperature was developed for further analysis.

The DMA Q800 measured the acting force and the elastic deflection on the center of the beam. The calculated Young’s modulus is based on analytical Equation (1):

(1) $$E=\frac{FL^{3}}{48\delta I}$$

where *E* is Young’s modulus at the specific temperature, *F* is the force acting on the center of the beam, *L* is the length of the beam, *δ* is the deflection at the midpoint, *I* is the area moment of inertia of cross-section which equals (bh^3^)/12 for specimen geometry.

Young’s modulus was also measured from natural frequency. Ten impact tests were conducted on uncracked beams at different temperature values ranging from 25 °C to 70 °C as per Figure 1. The natural frequency was recorded by a laser vibrometer. Young’s modulus was calculated from Equation (2):

(2) $$E={\left(\frac{2\pi }{\beta_{1}{}^{2}}\right)}^{2}\frac{\rho AL^{4}f_{1}{}^{2}}{I}=3.1934\frac{\rho AL^{4}f_{1}{}^{2}}{I}$$

where *E* is Young’s modulus, $\beta_{1}$ is the coefficient for the first first-order model with the boundary condition of the cantilever beam, ρ is the density of the material, *L* is the length of the beam, *f*_1_ is the natural frequency for the first mode, and *I* is the second moment of inertia.

As shown in Figure 1, 150 specimens, with an initially seeded crack ranging from 0 to 2.5 mm in depth, were tested on different temperatures and crack location values. For these specimens, at first, the impact test was conducted to measure a rough natural frequency range via laser vibrometer. Later, the shaker was set to run with a frequency sweep from 0 Hz to achieve the first-order natural frequency of the beam. Meanwhile, the accelerometer and the camera recorded the frequency and the amplitude responses, respectively. As the camera and accelerometer started recording at the same time, the actual natural frequency was found when the specimen showed the highest amplitude in the recorded video.

In contrast to the above tests, 30 specimens with an initial seeded 0.5 mm crack depth were tested on different temperatures and crack locations, as shown in Figure 1. At the start of each experiment, a fresh specimen with predefined crack depth was mounted on the shaker. An impact test was carried out to experimentally determine the first mode of the natural frequency of a fresh specimen by using the laser vibrometer. Later, the specimen was set to run at an operating frequency using the signal generator. Initially, this operating frequency was equal to the fundamental frequency obtained from the impact test.

The root mean square (RMS) value of the acceleration, with the help of the accelerometer, NI DAQ card, and Signal Express, was monitored once the specimen was excited on its first mode of natural frequency. A drop in the RMS value was used as a sign of change in the natural frequency due to the crack growth in the specimen. At this instant, the shaker was stopped, and crack depth measurements were taken with the camera. Later, the impact test was carried out again with a light wooden mallet to find the new natural frequency. This new frequency was then again used to excite the specimen to observe crack growth or propagation. This procedure was repeated until the catastrophic failure of the specimen. The failure of the specimen was defined as a point at which the specimen showed no amplitude at the free end.

### 2.2. Analytical Method

In this section, re-arrangement of existing analytical formulations are shown to express the effect of crack depth on the global dynamic response of a beam structure, while crack is assumed as a torsional spring. The natural frequency of a beam structure with a fixed-free configuration during testing can be expressed in the form of a Timoshenko beam. The analytical expression is given below (S. Rao [28]):

(3) $$f_{n}=0.5596\sqrt{\frac{EI}{\rho Al^{4}}}$$

where $f_{n}$ is the natural frequency in Hz, ρ is the density of the specimen, *E* is the elastic modulus, *I* is the moment of inertia, and A is the cross-sectional area. The factor of boundary condition (β l) for the first mode of the fixed-free position cantilever beam is provided by Rao, which is equal to 1.875104.

Equation (3) can be used to find the natural frequency of a fixed-free cantilever beam without crack or damage. Therefore, the analytical formulation is required to indicate the influence of the crack. Majid et al. [29] developed a formula that represents the natural frequency of a cracked beam:

(4) $$f_{nc}=f_{n}-\Delta f_{nc}$$

where:

$f_{nc}$ = natural frequency of the cracked beam,
$\Delta f_{nc}$ = difference between the natural frequencies of a cracked and un-cracked beam.

As shown in Figure 2, the crack of the selected non-prismatic cantilever beam could be modeled as a massless torsional spring. The spring stiffness established by Ostachowicz et al. [30] is shown in the following Equation (5).

(5) $$k_{t}=\frac{Ebh^{3}}{72\pi f\left(\frac{t_{c}}{h}\right)}$$

where *b* is the width of the beam, h is the thickness, $t_{c}$ is the crack depth, $f\left(\frac{t_{c}}{h}\right)$ is a crack function that can be found from Equation (6), also provided by Ostachowicz et al.:

(6) $$f\left(\frac{t_{c}}{H}\right)=0638{\left(\frac{t_{c}}{H}\right)}^{2}-1.035{\left(\frac{t_{c}}{H}\right)}^{3}+3.720{\left(\frac{t_{c}}{H}\right)}^{4}-5.177{\left(\frac{t_{c}}{H}\right)}^{5}+7.553{\left(\frac{t_{c}}{H}\right)}^{6}\;-7.332{\left(\frac{t_{c}}{H}\right)}^{7}+2.491{\left(\frac{t_{c}}{H}\right)}^{8}$$

Behzad et al. [31] re-arranged Equations (3)–(5), to have a comprehensive formula that can represent the crack depth and location with the material properties, as shown in Equation (7).

(7) $$f_{nc}=\left\{1-\left[\frac{72\pi \;I\;F\left(\frac{t_{c}}{H}\right)}{BH^{2}L}{\left(Cos\frac{\pi x}{2L}\right)}^{2}\right]\right\}\;f_{n}$$

The above formula can express the natural frequency of a cantilever beam in which the first part is indicating the crack effect as a fraction of the natural frequency $f_{n}$.

Input Equation (3) in Equation (7):

(8) $$f_{nc}=\left\{1-\left[\;\frac{72\pi \;I\;F\left(\frac{t_{c}}{H}\right)}{BH^{2}L}{\left(Cos\frac{\pi x}{2L}\right)}^{2}\;\right]\;\right\}\;\left[\;0.5596\sqrt{\frac{E.I}{\rho .A.l^{4}}}\;\right]$$

where *x* is the crack location, *L* is the length of the beam from the fixed point, ρ is the density of the ABS, and *I* is the moment of inertia.

### 2.3. Numerical Simulation

**Figure 4:**
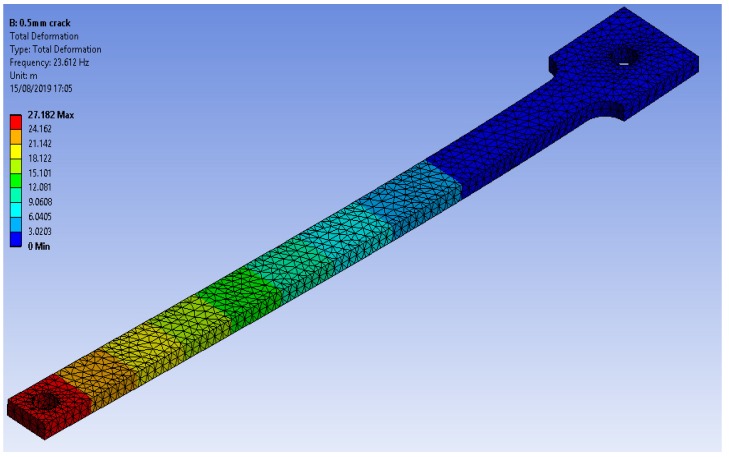
Modal analysis of the specimen for the first mode.

Modal analysis was conducted to observe the behavior of the structure with respect to different crack depth and locations using ANSYS © WorkBench v19.1, as shown in Figure 4. The geometry of the model was imported from SolidWorks©.

The boundary condition was a displacement that is set to be zero in all directions at the fixed end. The thermal load could not be applied in modal analysis; therefore, E of ABS at each temperature was set manually in the material properties field. Young’s modulus at elevated temperatures was found from the DMA, as explained in the next section. The mesh size was set to be 0.001 mm to have accurate natural frequency values.

## 3. Results

### 3.1. Young’s Modulus of 3D Printed ABS

**Figure 5:**
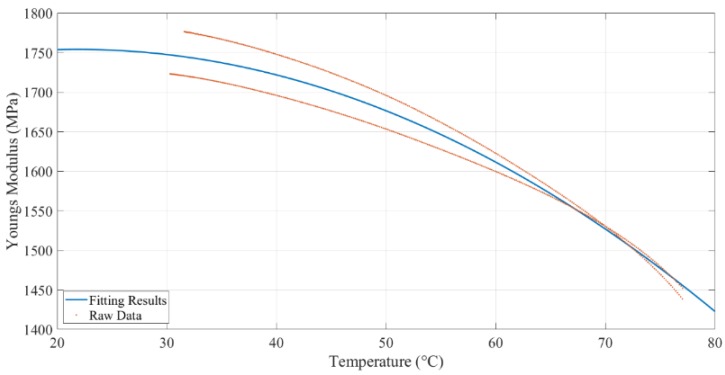
Extended DMA (dynamic mechanical analyzer) curve found by MATLAB.

Two specimens were tested by DMA. The results are shown in Figure 5. The poly2 curve fitting method ($y=ax^{2}+bx+c$) was selected to build the correlation between the temperature and Young’s modulus (storage modulus for elastic material). This curve fitting function is based on the least-squares method. The fitted results are shown in Equation (9) and Figure 5. The poly2 fit type showed a 95.29% R-square value. Calculated Young’s modulus, based on DMA tests, at different temperatures, are shown in Table 2.

(9) $$E=-0.09801T^{2}+4.286T+1701$$

where *E* is Young’s modulus (MPa), *T* is the temperature (°C).

**Figure 6:**
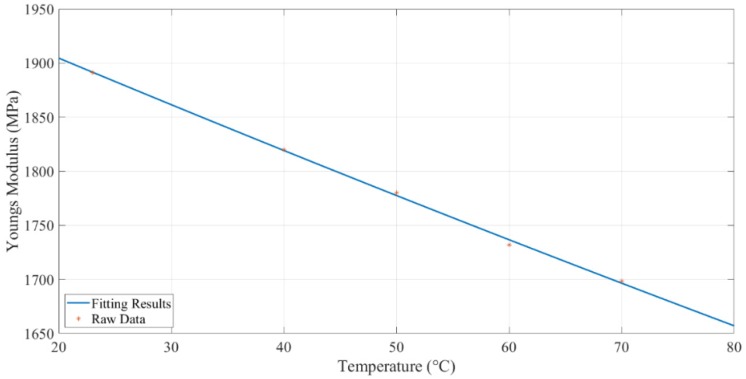
*E*-modulus curve found based on the natural frequency.

Similar to the bending test results, based on natural frequency, the empirical correlation between temperature and Young’s modulus was developed via curve fitting with the poly2 fit type. The fitting result is shown in Equation (10) and Figure 6. The poly2 fit type showed a 99.84% R-square value. Calculated Young’s modulus, based on natural frequency, at different temperatures, are shown in Table 2.

(10) $$E=0.003001T^{2}-4.417T+1991$$

### 3.2. Analytical and Numerical Results

**Figure 7:**
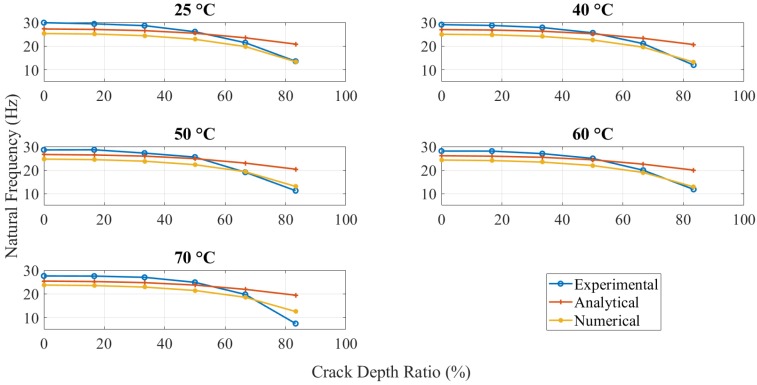
Natural frequency for different crack depths and temperatures at the crack location of 5 mm.

Apart from the experimental tests, the analytical and numerical values of the natural frequency of the ABS beam with different configurations were calculated, as shown in Figure 7. This plot presents the tendency and difference between the experimental, analytical, and numerical results.

The natural frequency of ABS specimens, with different crack depth values and location tested at five elevated temperatures, was calculated analytically (as per Equation (8)), numerically, and experimentally. The same trend, as shown in Figure 7 for all approaches, proved that ABS polymers behaved normally with respect to crack depth and temperature. As expected, specimens with greater crack depths showed a less natural frequency, and the same behavior was observed for applied temperatures.

**Figure 8:**
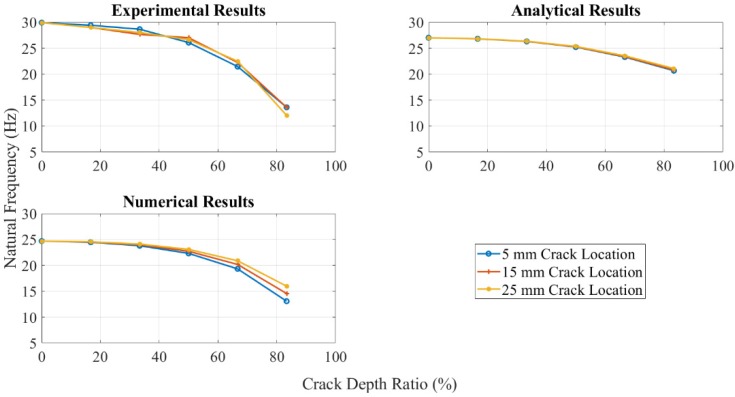
Natural frequency change for crack location by three methods at 25 °C.

Figure 8 shows the effect of the crack location on the natural frequency. The numerical results showed that the crack close to the fixed end had more obvious natural frequency change when altering the crack depth. While the natural frequency difference between various crack locations increased as the crack was distant from the fixed end. The behavior of ABS polymer at 15 mm and 25 mm crack locations was the same as when the crack location was at 5 mm, i.e., the natural frequency decreased with increasing crack depth and temperature.

### 3.3. Dynamic Response Results of Initially Seeded Crack without Propagation

**Figure 9:**
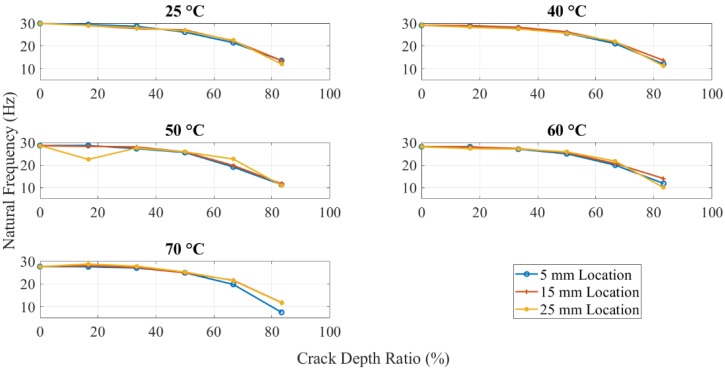
Natural frequencies of different crack depth beam for the crack at different locations.

Figure 9 shows the natural frequencies of the same crack depth beam with different temperatures and crack locations. Theoretically, the 25 mm crack location supposed to have a high natural frequency than the other 5 mm and 15 mm. Because the same crack depth can cause the same decrease in local stiffness, the crack location near the fixed end leads to a high bending moment and results in high-frequency drop. However, the experimental results observed extremely close values. The natural frequency of 25 mm location crack was observed lower than other locations when crack depth was about 2.5 mm. This was considered as a possible error due to difficulties in capturing the dynamic response experimentally when crack depth to specimen thickness was high. As per the test observations, when the crack depth was more than 80% of the specimen thickness, the response of the structure was disturbed because of the obvious bend along the beam. The overall behavior of the 3D printed ABS beam was as expected. An increase in crack depth and temperature showed a decrease in the global natural frequency of the structure due to a decrease in beam stiffness. The crack location had a relatively minor influence. Beams with cracks near to the fixed end had less natural frequency value compared to when the cracks were located at a distance.

**Figure 10:**
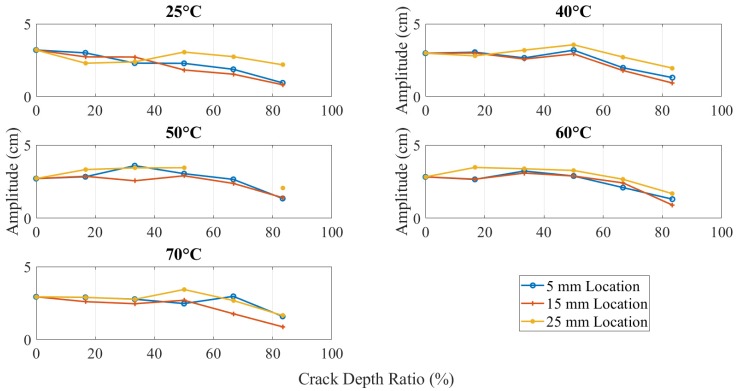
The amplitude of different crack depth beam for the crack at different locations.

The amplitudes for different crack locations with various temperatures are plotted in Figure 10. All curves were found to have declined trend. Although there were some fluctuations during the crack propagation when crack depth was more than 1.5 mm, amplitude decreased on high values of crack depth. Moreover, the relationship was inversely proportional between amplitude and temperature, as heated specimens had a lower amplitude than the unheated ones.

**Figure 11:**
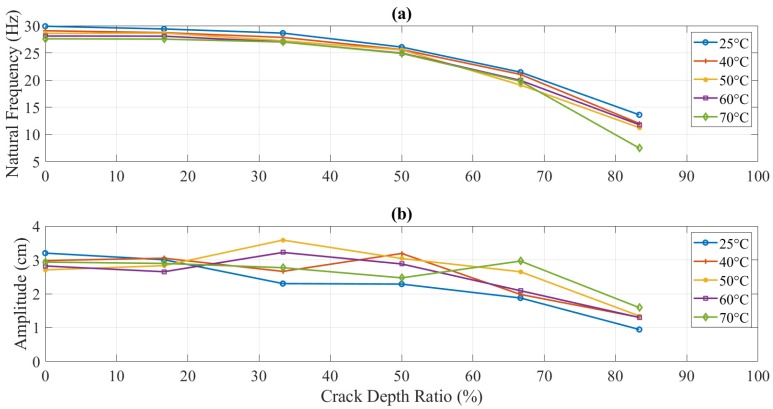
Natural frequency (**a**) and amplitude (**b**) of the initially seeded-crack experiment at different temperatures of 5 mm crack location.

Figure 11 shows the effect of temperature variation on crack depth values located at 5 mm from the fixed end. The natural frequency of the structure was reduced with increased temperature for the same amount of damage. However, the amplitude change was observed as random. The same behavior for the other two locations was noticed in terms of natural frequency and amplitude. On high values of temperature and crack depth, the natural frequency showed a decrease. The amplitude showed an overall reduction against crack depth and temperature values. However, there was an inconsistency in the behavior that is discussed in the next section.

### 3.4. Dynamic Response Results of Propagating Crack

**Figure 12:**
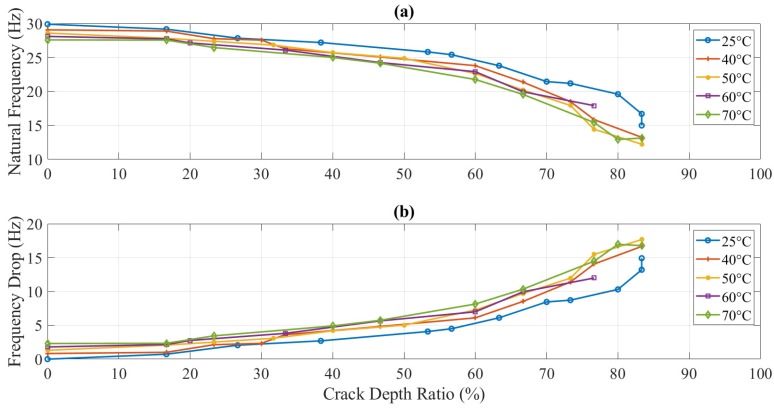
Natural frequency (**a**) and frequency drop (**b**) of the propagating-crack experiment at different temperatures of 5 mm crack location.

The trends of natural frequency and amplitude responses during crack propagation were somehow similar to non-propagating tests, as mentioned above. Figure 12 plots the frequency drops during crack propagation at a 5 mm location. Due to a decrease in stiffness with propagating crack, the natural frequency was also decreased. However, the difference due to temperature was not obvious. Both crack depth and the temperature had an independent influence on the dynamic response as the first one was amplified with the increment of the latter one.

**Figure 13:**
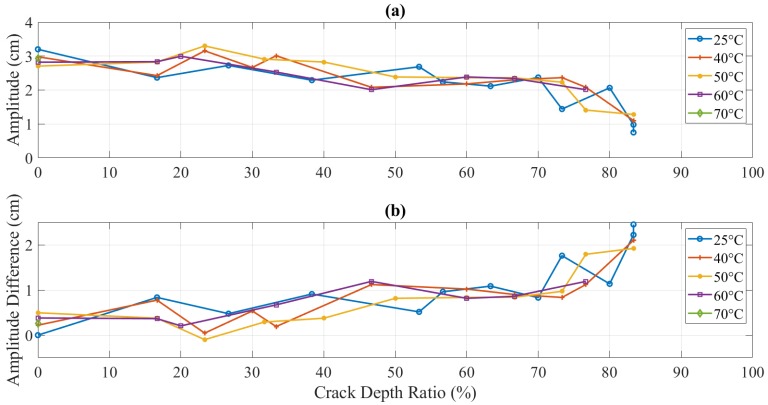
Amplitude (**a**) and amplitude difference (**b**) of the propagating-crack experiment at different temperatures of 5 mm crack location.

The amplitude change during crack propagation is plotted in Figure 13. However, the experimental data was extremely stochastic. Furthermore, an overall decreasing trend was observed for amplitude with an increase in crack propagation. It was contrary to analytical results, as mentioned before.

### 3.5. Empirical Correlation

**Figure 14:**
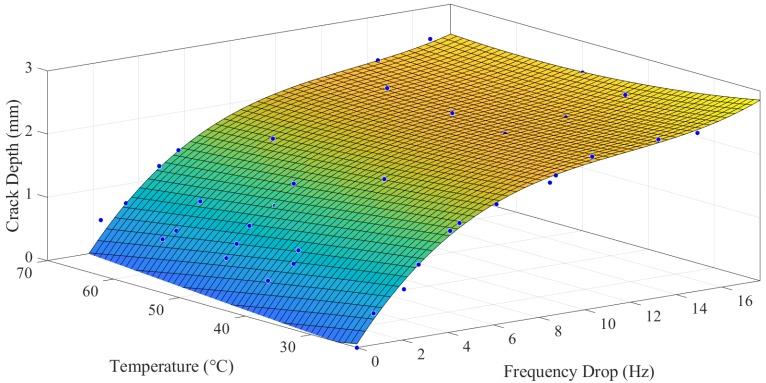
Experimental empirical correlation based on natural frequency for a crack location of 5 mm from the fixed end. Note: The plotted crack depth also includes the initial seeded crack depth value.

Based on the above-observed data, for specimens with a crack location at a distance of 5 mm from the fixed end, a surface was plotted between temperature values, frequency drop, and crack depth, as shown in Figure 14.

Poly32 function was applied for surface fitting. The R-square value of this fit was calculated as 98.51%. The model function is shown in Equation (11):

(11) $$t_{c}=f\left(\Delta \omega_{nc},T\right)$$

$$\begin{array}{l} t_{c}=0.4355+0.4689\Delta \omega_{nc}-0.01418T-0.0359\Delta \omega_{nc}{}^{2}\\ \;+0.0008476\Delta \omega_{nc}T-9.305\times 10^{-5}T^{2}\\ \;+0.001083\Delta \omega_{nc}{}^{3}-0.000111\Delta \omega_{nc}{}^{2}T\\ \;+2.158\times 10^{-5}\Delta \omega_{nc}T^{2} \end{array}$$

where Δ*ω_nc_* is the frequency drop, and *T* is the temperature. Similarly, the correlation for 15 mm and 25 mm crack locations was also established. The integrated model for all the correlations is shown in Equation (12). The coefficients of Equation (12) are shown in Table 3.

(12) $$\left[\begin{array}{c} t_{5}\\ t_{15}\\ t_{25} \end{array}\right]=\left[\begin{array}{ccc} A_{5} & B_{5} & C_{5}\\ A_{15} & B_{15} & C_{15}\\ A_{25} & B_{25} & C_{25} \end{array}\right]\left[\begin{array}{c} 1\\ \Delta \omega_{nc}\\ T \end{array}\right]+\left[\begin{array}{ccc} D_{5} & E_{5} & F_{5}\\ D_{15} & E_{15} & F_{15}\\ D_{25} & E_{25} & F_{25} \end{array}\right]\left[\begin{array}{c} \Delta \omega_{nc}{}^{2}\\ \Delta \omega_{nc}T\\ T^{2} \end{array}\right]\;+\left[\begin{array}{ccc} G_{5} & H_{5} & I_{5}\\ G_{15} & H_{15} & I_{15}\\ G_{25} & H_{25} & I_{25} \end{array}\right]\left[\begin{array}{c} \Delta \omega_{nc}{}^{3}\\ \Delta \omega_{nc}{}^{2}T\\ \Delta \omega_{nc}T^{2} \end{array}\right]$$

## 4. Discussion

### 4.1. Young’s Modulus Determination

Young’s modulus of 3D printing ABS material at different temperatures was measured by two methods. The theoretical Young’s modulus for 3D printed ABS specimen with 90% infill parameters was calculated as 1618.5 MPa. As we used 100% infill density for printing to get the best strength, we assumed Young’s modulus was proportional to the infill density, so

(13) $$E=\frac{1618.5}{90\%}=1798.33\;MPa$$

**Figure 15:**
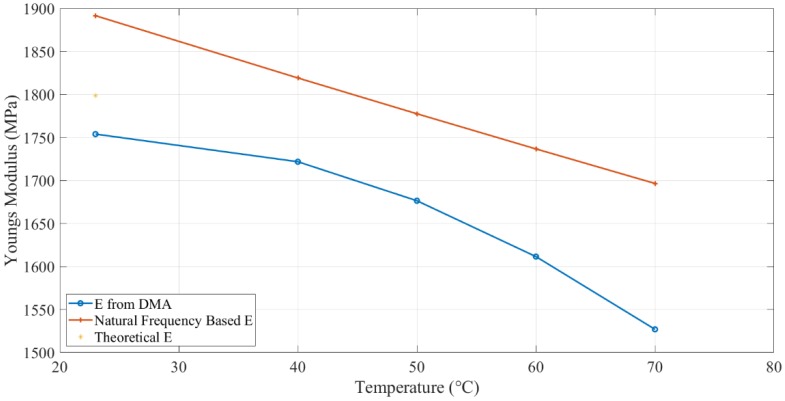
E-modulus by DMA and calculation.

The theoretical Young’s modulus for 3D printing ABS used in the tests was 1798.33 MPa. It was the same as the *E* value at 25 °C (1753.88 MPa), which was measured by DMA, as shown in Figure 15.

The Young’s modulus results calculated by the Timoshenko beam equation (Equation (8)) were higher than DMA results. It looks unreasonable because Young’s modulus is a mechanical property that is supposed to be constant at a specific temperature. This difference could be due to several reasons. Young’s modulus measured by both tests, as a global parameter, was related to the torsional stiffness of the structure, as shown in Equation (14).

(14) $$E=\;\frac{2\left(1+v\right)kL}{J}$$

where *v* is Poisson Ratio, k is the torsional stiffness, *L* is the length of the element, and *J* is the torsion constant for the section.

The environment temperature could affect the torsional stiffness. As the air in the chamber was heated during the DMA test, it might have reduced the whole structural stiffness. However, the heating mats only heated the area near the fixed end, and hence reduced the local stiffness only, as shown in Equations (15) and (16):

(15) $$E_{DMA}=\;\frac{2\left(1+v\right)k_{T}L}{J}$$

(16) $$E_{f}=\;\frac{2\left(1+v\right)k_{T}l}{J}+\frac{2\left(1+v\right)k_{RT}\left(L-l\right)}{J}$$

Therefore, Young’s modulus from the Timoshenko beam equation $E_{f}$ was higher than the DMA results *E_DMA_*. On the other hand, because the heat mats were placed near to the fixed end of the beam, not only the adhesion force between them and the beam increased the little local stiffness but also the mass of the heat mats influenced the structural E-modulus seriously, as shown in Equation (17):

(17) $$E={\left(\frac{2\pi }{1.875104^{2}}\right)}^{2}\frac{AL^{3}f_{1}{}^{2}\left[\rho_{ABS}L+\left(\rho_{mean}-\rho_{ABS}\right)l\right]}{I}$$

The Young’s modulus of the beam with heating mats was high than the one without heat mats due to an increase in the overall structural density. The above reasons led to the difference between Young’s modulus. As the heating mats were always on the beam, Young’s modulus from the Timoshenko beam equation was applied as the whole system’s Young’s modulus in the rest of the experiments.

### 4.2. Dynamic Response

A comparison of the natural frequency results from all the methods is shown in Figure 7. The specimen with high crack depth showed a lower natural frequency value than the specimen with less crack depth. This change in natural frequency value could be related to the reduction in the stiffness of the structure caused by the crack. The crack created a localized effect at the crack tip in which the stress concentration was accumulated, suggesting that the stress concentration at the crack tip was increased with an increase in the crack depth to thickness ratio. All the specimens showed the same behavior for different crack depths and locations at different values of temperature.

In the numerical simulation, the modal analysis fairly estimated the natural frequency of anisotropic structure resembling the fabricated 3D printed ABS polymers. The specimen was an extrusion-based fabrication with layers, and the properties were not only dependent on the intrinsic properties but also on the printing parameters. However, the validity of the numerical simulation, especially at lower crack depth, was related to the fill density of print since it was set to be 100% while printing. The high filling density of the ABS filaments facilitated close-to-isotropic behavior that estimated close natural frequency values in experimental methods.

The analytical model (Equation (8)) was found to be valid and useful for anisotropic materials, such as ABS polymer. From Figure 7, a good agreement was observed between experimental and analytical results as the difference was less than 10%. The analytical natural frequencies were always observed higher than the numerical results for different crack depth. However, the difference between both was increased from around 2 Hz to 7 Hz gradually as the crack depth increased. The limitation of the crack function $f\left(\frac{t_{c}}{h}\right)$ used in the analytical model caused this gradually increasing difference. It overestimated the value when the crack depth was large. Therefore, the natural frequency of the analytical model had an increasing error on high crack depth values.

In experiments, the natural frequency was measured higher than other methods when the crack was smaller. Because heating mats were mounted on the beam, they made the overall structure stiffer and, hence, increased the natural frequency. However, it was decreased very quickly when crack depth was increased but observed close to other methods at 80% crack depth to thickness ratio.

**Figure 16:**
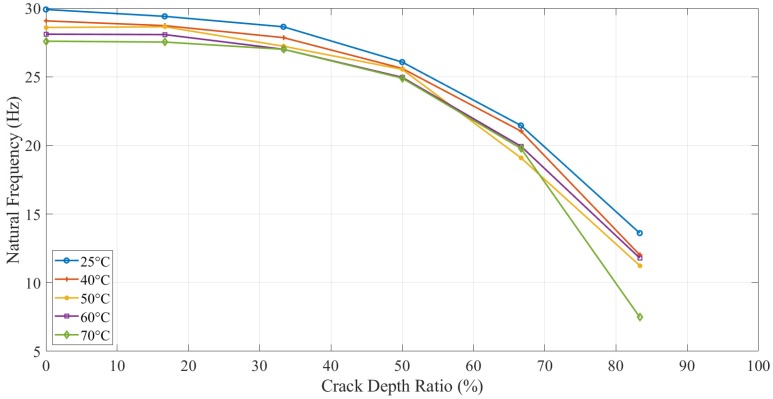
The experimental natural frequency of pre-initiated crack at 5 mm location.

**Figure 17:**
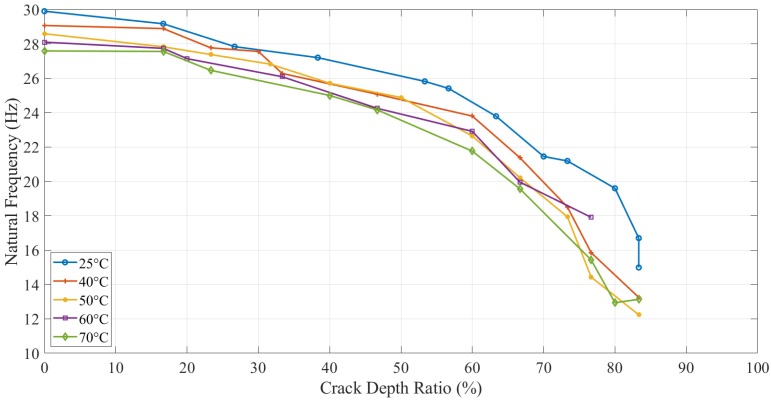
The experimental natural frequency of propagating crack at 5 mm location.

In this study, dynamic response (i.e., natural frequency and amplitude) was used to determine the parameters of the crack (i.e., mainly crack depth) but in the presence of different values of temperature. Figure 16 and Figure 17 illustrate the behavior of the natural frequency of 3D printed ABS beam at different crack depths and locations exposed to elevated temperatures. The trends showed a regular pattern. In terms of temperature, a specimen at 25 °C showed a natural frequency higher than the one that was at 40 °C. E-modulus values showed a decrease as the testing temperature was increased, which means that the natural frequency of the structure was influenced by an increase in the thermal load at the same amount of damage. Increasing the temperature of any structure was considered as unhealthy since it would decrease the E-modulus that subsequently decreases the natural frequency, which can allow the structure to resonate more likely at low cyclic load, resulting in serious damages. This behavior was observed in this study for both types of tests, i.e., with and without crack propagation tests. Propagating-crack tests were found more accurate due to their instant measurements throughout the crack growth. The width of the propagated crack was relatively small and bit genuine as compared to the seeded cracks. This width initiated at the base of the given seeded crack and showed a more representative decrease in natural frequency due to real propagation.

**Figure 18:**
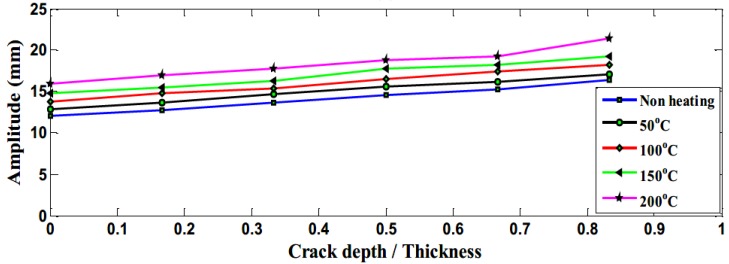
The experimental amplitude of aluminum experiment at the crack location of 5 mm tested at elevated temperature [31].

**Figure 19:**
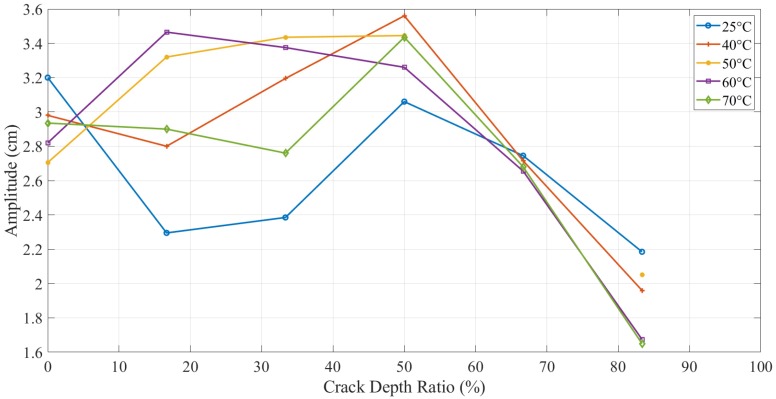
The experimental amplitude of pre-initiated crack at 25 mm location.

**Figure 20:**
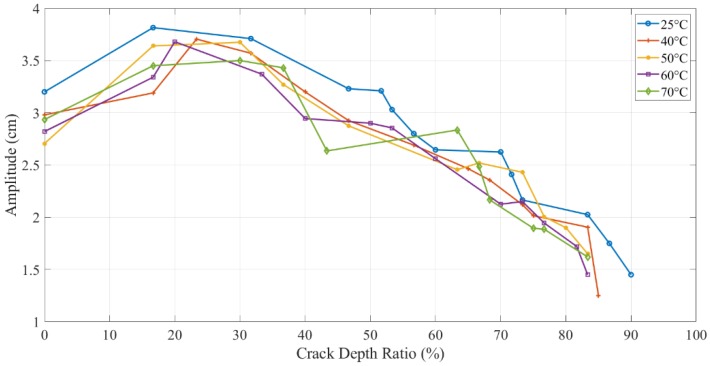
The experimental amplitude of propagating crack at 25 mm location.

Theoretically, the amplitude should increase as the crack depth and temperature increase due to the reduction in the structural stiffness, as observed by the authors as shown in Figure 18 [31]. On the other hand, the amplitude discrepancy, illustrated in Figure 19 and Figure 20, was observed for crack depth and temperature. Both types of tests showed random amplitude values; however, the overall behavior showed a decrease in amplitude with an increase in crack depth and temperature.

The amplitude behavior was consistent to some extent when the crack was at a distance from the fixed end because the bending moment was minimum. Figure 20 shows amplitude versus crack depth at different temperatures located at 25 mm from the fixed end. An increasing trend was observed at low values of crack depth to thickness ratio.

**Figure 21:**
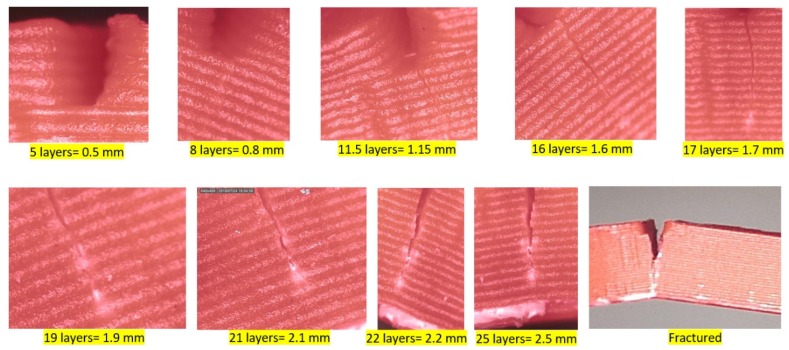
Crack propagation throughout the specimen from the microscope.

Some more observations were captured to justify the random amplitude behavior while monitoring the crack propagation. Figure 21 shows the magnified images of crack propagation in steps from the initial seeded crack depth of 0.5 mm to catastrophic fracture.

**Figure 22:**
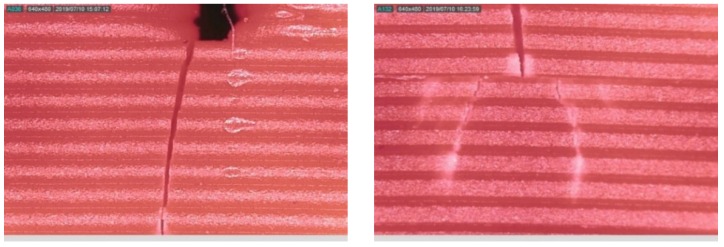
(**Left**) Propagated crack width compared with fabricated crack; (**Right**) Crack separation during propagation.

The width of the propagated crack compared to the initially seeded one (0.5 mm) was very small, as seen in Figure 22. After this crack initiation, the crack tip was no longer a rectangular shape and propagated across the thickness of the specimen either with a continuous or discontinuous contour, as shown in Figure 22 (left) and (right), respectively.

There were two prominent reasons, which might be the root causes of the observed stochastic amplitude of the dynamic response. The first was the anisotropic nature of 3D printed ABS. The printed specimen had various layers that were intact due to adhesion. But each of these layers might have slightly different material properties due to fusion and cooling at the time of printing. This slight difference could allow the seeded crack to propagate in a non-linear and/or irregular pattern and, hence, led to establishing a non-linear stiffness in the beam around the zone of crack propagation. This non-linearity in the beam stiffness ultimately caused a stochastic influence on the observed dynamic response. However, we observed a consistency natural frequency response during the test, which showed that the amplitude of the beam was highly sensitive even to a small non-linearity in the beam stiffness, while the latter didn’t affect much the natural frequency of the beam.

The second reason that could justify the inconsistency in the amplitude response was the phenomenon of stress crazing. It is a very popular phenomenon in polymers that induces the rearrangement of molecules of the ABS, creating micro-voids, and facilitate energy dissipation and, therefore, the crack propagation. Similar crazes in the ABS have also been reported in a previous study while monitoring the crack with a microscope [32]. The observed crazing was slightly nonlinear, as shown in Figure 22, and, hence, led to a non-linearity in beam stiffness at a small scale, further supporting our argument, as mentioned in the above para.

While monitoring the crack propagation through the specimen, the crack was propagating layer by layer. Each layer was observed as a barrier in front of the crack. Hence, a decrease in the layer thickness would increase the number of layers (barriers) and, subsequently, the crack resistance. This conclusion was also discussed by Rabbi et al., 2019 [33] that the crack requires high energy to propagate through the filaments. Moreover, the temperature of the nozzle could be increased in order to maintain high adhesion between the layers and to arrest the cracks between the layers.

The empirical correlation based on surface fits, as detailed in Equations (11) and (12), was based on crack-propagation tests. The natural frequency trends were consistent, and we established the correlation with very high accuracy of fit, as shown in Table 4.

Surface fits based on amplitude could not be established as the dynamic response was disturbed and irregular, as detailed in the previous section. For the reasons mentioned, amplitude behavior in 3D printed ABS was treated ineligible to be correlated and utilized as a damage assessment tool, at least, in the current experiment and specimen conditions. The “stress-crazing” led to a discrepancy in the amplitude behavior. The empirical correlation based on the amplitude has been found difficult to be established for polymers, unlike metals [34].

In this study, the existence of a crack changed the material properties of the specimen that ultimately caused the drop in its natural frequency to catastrophic failure. Mathematically, the empirical correlation obtained from this trend could be applied to estimate the crack depth if the frequency drop and temperature are known. The obtained correlation could be very useful for in-situ damage assessment of 3D printing ABS structures. At any instant of time, one just needs a frequency drop and temperature value to determine the crack depth and assessing the structural integrity and performance.

## 5. Conclusions

The dynamic response of non-prismatic 3D printed ABS cantilever beam was tested in a fixed-free position under thermal-mechanical load. The specimen was mounted on a shaker and excited at its natural frequency with different values of crack depth and temperature. Analytical, numerical, and experimental methods were used to observe the natural frequency and amplitude responses. A good match between the results and observations were observed for natural frequency response. The DMA results showed a decrease in E-modulus with an increase in temperature. The reduction in E-modulus was about 12% from 25 °C (i.e., room temperature) to 70 °C and led a drop in natural frequency in a close agreement with the obtained analytical and numerical results. It was also found that existing analytical formulation for crack beams could be applied for an anisotropic material, such as 3D printed ABS.

Tests concluded a decreasing natural frequency response with an increase of crack depth values. This behavior was observed more rigorously in the presence of high temperatures. The natural frequency response showed more influence of temperature than the crack depth at lower values of crack depth to thickness ratio. But a significant dependency was observed on crack depth at higher values of this ratio. Moreover, the influence of crack location was observed, as expected. The natural frequency at the same amount of damage showed a decreasing trend when the crack was located near to the fixed end. This trend was associated with the bending moment exerted by the length of the beam on the crack where stress was concentrated and, hence, led to a drop in natural frequency.

Crack propagation was monitored during continuous excitation. The filament layer was found as a barrier ahead of the crack. This was due to the high energy required by the crack to propagate through the filament. A decrease in the layer thickness would lead to an increase in the number of layers at the same thickness and, hence, could enhance the crack resistance.

Unlike the regular trend of natural frequency, the amplitude showed inconsistent behavior. The amplitude of the dynamic response of the specimen showed less dependency on the intrinsic properties of ABS. A random amplitude behavior in the crack propagation tests was observed, i.e., increase in the start and then decrease from half of the way. Stress crazing in polymers was concluded as a reasonable justification for this random trend. The crazes at the crack tip were induced due to a random rearrangement of molecular orientation and caused a permanent deformation, which led to a change in the material properties and, therefore, the amplitude response.

The empirical correlation was established for three crack locations, and their coefficients were found by applying a polynomial equation. This correlation could be utilized as an in-situ damage assessment tool for ABS 3D printed structures. The prediction of the crack depth could be implemented using natural frequency drop, crack location, and temperature as inputs. The fitting accuracy based on the results was found more than 92% for all crack locations. The empirical correlation based on stochastic amplitude response was found difficult to develop.

## Figures and Tables

**Table 1:** 3D printing parameters that are used for producing the specimens.

Parameter	Value
Nozzle size	0.4 mm
Layer height	0.1 mm
Infill density	100%
Print orientation	±45°
Print speed	45 mm/s
Extruder temperature	235 °C
Bed temperature	75 °C
Wall thickness	1.05 mm

**Table 2:** E-modulus of 3D printed ABS at an elevated range of temperature.

Temperature (°C)	RT (25)	40	50	60	70
Fitted *E* (MPa) based on DMA model	1753.88	1721.76	1676.41	1611.45	1526.89
Experimental average natural frequency (Hz)	28.24	27.70	27.40	27.03	26.76
Experimental *E* (MPa) based on natural frequency	1890.34	1819.78	1780.23	1731.80	1698.51
Fitted *E* (MPa) based nn fatural Frequency	1890.91	1819.04	1777.57	1736.71	1696.45

**Table 3:** Calculated empirical correlation coefficients.

Coefficients	Crack Location		
	5 mm	15 mm	25 mm
A	0.4355	1.199	0.5384
B	0.4689	0.4678	0.3923
C	−0.01418	−0.05725	−0.0234
D	−0.0359	−0.03507	−0.02931
E	0.0008476	0.001446	0.00266
F	−9.305 × 10^−5^	0.0004688	2.049 × 10^−5^
G	0.001083	0.0009888	0.000847
H	−0.000111	−4.528 × 10^−5^	−0.0001377
I	2.158 × 10^−5^	−4.272 × 10^−5^	6.593 × 10^−5^

**Table 4:** The accuracy of the surface fits in the form of R-square value.

Surface Fit	R-Square
Crack at 5 mm location	0.9851
Crack at 15 mm location	0.9614
Crack at 25 mm location	0.9230
